# Semi-automated screening of biomedical citations for systematic reviews

**DOI:** 10.1186/1471-2105-11-55

**Published:** 2010-01-26

**Authors:** Byron C Wallace, Thomas A Trikalinos, Joseph Lau, Carla Brodley, Christopher H Schmid

**Affiliations:** 1 Department of Computer Science, Tufts University, Medford, MA, USA; 2 Center for Clinical Evidence Synthesis, Institute for Clinical Research and Health Policy Studies, Tufts Medical Center, Boston, MA, USA; 3 Biostatistics Research Center, Institute for Clinical Research and Health Policy Studies, Tufts Medical Center, Boston, MA, USA

## Abstract

**Background:**

Systematic reviews address a specific clinical question by unbiasedly assessing and analyzing the pertinent literature. Citation screening is a time-consuming and critical step in systematic reviews. Typically, reviewers must evaluate thousands of citations to identify articles eligible for a given review. We explore the application of machine learning techniques to semi-automate citation screening, thereby reducing the reviewers' workload.

**Results:**

We present a novel online classification strategy for citation screening to automatically discriminate "relevant" from "irrelevant" citations. We use an ensemble of Support Vector Machines (SVMs) built over different feature-spaces (e.g., abstract and title text), and trained interactively by the reviewer(s).

Semi-automating the citation screening process is difficult because any such strategy must identify all citations eligible for the systematic review. This requirement is made harder still due to class imbalance; there are far fewer "relevant" than "irrelevant" citations for any given systematic review. To address these challenges we employ a custom active-learning strategy developed specifically for imbalanced datasets. Further, we introduce a novel undersampling technique. We provide experimental results over three real-world systematic review datasets, and demonstrate that our algorithm is able to reduce the number of citations that must be screened manually by nearly half in two of these, and by around 40% in the third, without excluding any of the citations eligible for the systematic review.

**Conclusions:**

We have developed a semi-automated citation screening algorithm for systematic reviews that has the potential to substantially reduce the number of citations reviewers have to manually screen, without compromising the quality and comprehensiveness of the review.

## Background

In this section we first motivate our work by presenting a brief overview of the systematic review process in general, and the abstract screening component in particular. We then review previous research in biomedical text classification, which provides the foundation for our contribution.

### On Systematic Reviews

Systematic reviews (with or without meta-analysis) are increasingly used to inform all levels of healthcare, from bedside individualized decisions to policy-making. Like all scientific approaches, a systematic review tries to address a well-formulated research question by following a protocol of well-defined steps [1,2]. To minimize selection bias, systematic reviews appraise and analyze all research reports that fulfill a set of pre-defined eligibility criteria. To identify all eligible reports, reviewers conduct broad searches of the literature, and then manually screen the titles and abstracts of all returned citations. All relevant (potentially eligible) citations are retrieved and reviewed in full text to select those that are ultimately included in the systematic review.

Screening of citations for systematic reviews is a tedious and time-consuming, yet critical, step. Failure to identify eligible research reports threatens the validity of the review. Typically, reviewers screen between 2,000 and 5,000 citations, approximately 200 to 500 of which are deemed relevant and are reviewed in full text. From these, at most a few dozen are ultimately included in the systematic review. Much larger projects are not uncommon. For example, in a project that involved three evidence reports conducted for the United States Social Security Administration on the association of low birth weight, failure to thrive, and short stature in children with disability, the Tufts Evidence-based Practice Center screened over 33,000 abstracts [3-5].

An experienced reviewer can screen an average of two abstracts per minute. At this rate, a project with 5,000 abstracts requires 5 person days (40 hours) of uninterrupted work time. Abstracts for difficult topics may take several minutes each to evaluate, thus multiplying the total time needed to process them by several fold.

Herein, we modify the typical (manual) approach to screening citations for systematic reviews in order to semi-automate the process. We use a classification model to automatically exclude irrelevant citations. The reviewers will trust the model's exclusions, and will only screen those citations that are suggested by the classifier. The aim of our approach is to reduce the reviewers' workload, allowing them to focus on the more intellectually demanding steps of the systematic review (e.g., interpretation and analysis), while reducing costs.

### Previous Work

In this section we review related work. We first discuss previous applications of machine learning techniques to biomedical text classification. Next, we review some of the machine learning tools we use (Support Vector Machines and the active learning framework, in particular).

#### Previous Applications of Machine Learning to Biomedical Literature

Due to the exponential growth of available biomedical literature [6], much work has been done in automatically mining and learning from published manuscripts. Here we briefly review the emerging body of promising research on applications of machine learning methods to biomedical text classification [7-11], particularly those works focused on automatic classification of biomedical abstracts into clinically relevant categories.

Aphinyanaphongs et al. applied machine learning techniques to automatically discriminate "high-quality" from "low-quality" articles in the domain of internal medicine [10]. They explored classification using several different feature-spaces (this is sometimes referred to as *multi-view learning *[12]). A feature-space is the mathematical space where the points for each citation for a particular feature set live. For example, title text is a feature-space, and each citation is represented by exactly one point in this space, corresponding to the vector representation (e.g., bag-of-words encoding) of its title. They found that using publication type, abstract text, title text, and Medical Subject Headings (MeSH) terms as features with a Support Vector Machine (SVM) classifier resulted in the best performance.

Building on this work, Kilicoglu et al. demonstrated the feasibility of automatically identifying "scientifically rigorous" articles using classification algorithms [8]. They too found that using multiple features from publications, including "high-level" features such as Unified Medical Language System (UMLS) terms, boosted classification accuracy. Additional research [13-15] has further corroborated the observation that biomedical text classification can be improved by using multiple feature-spaces, and by enriching raw text with additional information (e.g., with automatically extracted UMLS terms, or via other Natural Language Processing techniques).

Most similar to the present work, Cohen et al. demonstrated that machine learning techniques can reduce the labor required to update systematic reviews [16]. In particular, they used a boosted perceptron-based classifier to predict when new articles should be added to existing drug class systematic reviews. (A perceptron is a type of neural network that finds a linear function to discriminate between classes.) They aimed to reduce the number of abstracts reviewers must manually peruse to update a systematic review while maintaining 95% sensitivity to new articles that ought to be added to the review. They experimented over 15 datasets. Over each of these they varied a key parameter - the false negative learning rate, *w *- over a range of values and reported the best achieved performance (i.e., results for the best observed value of *w*). They found that their approach could theoretically reduce the number of abstracts that needed to be evaluated to update systematic reviews by between 0.0 and 68%, while maintaining a sensitivity of 95% to the eligible citations. (Though in one dataset they were unable to achieve 95% sensitivity.)

The above works have demonstrated the potential of machine learning methods to mitigate the burden imposed by the overwhelming volume of published biomedical literature. The task of semi-automating citation screening is unique due in part to the obstacles outlined by Cohen et al. [16]. In particular, the requirement of identifying all of the eligible citations changes the goal of the classification task; rather than attaining high accuracy, as is usually the objective in classification, we aim to eliminate the need to review clearly irrelevant citations without wrongly excluding eligible ones. Our task differs from that delineated by Cohen et al. in that we aim to semi-automate the citation screening step while conducting systematic reviews, rather than semi-automating the process of *updating *previously conducted systematic reviews.

#### Active Learning with SVMs

As discussed above, reviewers conducting systematic reviews first search a database (e.g., PubMed) with a carefully constructed query tailored to the medical question being investigated. Next, they peruse and then categorize each of the returned abstracts as either "relevant" or "irrelevant" to the review. This latter step of determining which articles are suitable for inclusion is a laborious, time-intensive process. Moreover, the reviewers are typically physicians, and their time is therefore expensive. Thus, we have access to a large "pool" of unlabeled data (the citations retrieved via the database query) and an "oracle" (the reviewer) that can provide labels ("relevant", "irrelevant"'), at a cost. This scenario is exactly the sort that motivated the development of *pool-based active learning *[17], wherein the expert trains the classifier interactively by providing labels for instances the classifier "thinks" will be most informative.

The basic idea in active learning is that if the classifier is allowed to select the data with which it is trained, as opposed to passively accepting a training dataset, the training process can be expedited. Furthermore, it has been argued [18] that active learning over a small subset of informative data can actually produce a better generalized model than one trained over more, randomly selected data. For a recent survey of active learning, see Settle's literature review [19]. In this work we focus on pool-based active learning with Support Vector Machines (SVMs).

Briefly, SVMs are classifiers that work by finding a hyperplane that separates instances into their respective classes in feature-space [20]. SVMs use *kernel functions *to calculate the separating hyperplane in a computationally efficient manner, and can scale gracefully to problems with high-dimensional data (e.g., text). A kernel function returns the inner product between feature vectors mapped into a high-dimensional space. In many cases the inner product can be calculated without explicitly computing these higher dimensional feature vectors, allowing for fast, scalable computation. We use SVMs because they have empirically performed well over high-dimensional text data in general [21], and in the context of biomedical text classification in particular [9,10]. (In any case, the choice of classification algorithm seems to matter less than the choice of features when working with biomedical texts [22].)

Tong and Koller [23] presented an active learning strategy for SVMs, called SIMPLE, that works as follows. Given at least one labeled example from each class, construct an SVM (i.e., find an initial separating hyperplane). Next, ask the expert to label the (unlabeled) instance closest to the current hyperplane. The intuition is that examples near the hyperplane are those about whose label the classifier is most uncertain. Repeat this process until some stopping criterion is met (e.g., the expert refuses to provide any more labels). This method has been shown to work well empirically [18], and we use it as the foundation of our approach. We shall elucidate why SIMPLE must be tailored to the problem of citation screening in later sections. (We note that Tong and Koller also proposed two other active learning approaches for SVMs [23]; we use SIMPLE due its simplicity and empirical success.)

## Results

In this section, we first present our active learning strategy for biomedical citation classification. We then report results from experiments conducted over three previously conducted systematic reviews. We demonstrate that our technique can significantly reduce the burden on reviewers without excluding any relevant citations.

### Our Approach to Semi-Automating the Citation Screening Task

Our novel semi-automated citation screening strategy comprises two major components: our approach to document representation and our novel active text classification learning strategy. The latter includes the model training process, classification algorithm, ensemble method and sampling technique used. We also address the question of how many citations should be manually labeled before allowing the model to classify the remaining documents. We present these components in the following subsections, but first outline how we envision the semi-automated step fitting into the citation screening process.

**Figure 1:**
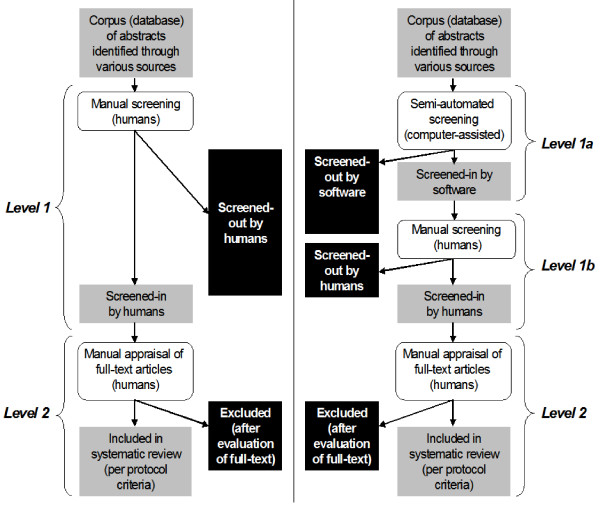
**Shown are the typical approach and our modified approach that includes semi-automated abstract screening on the left and right-hand, respectively (see text for details)**. In the modified approach the reviewers train and use a classification model to exclude completely "irrelevant" citations ("Level 1a"). They will trust the model's exclusions, and will review only the citations suggested by the classification model.

Figure 1 juxtaposes the typical screening process with our semi-automated approach. In the typical citation screening process, humans evaluate the whole set of citations in the dataset and select (or "screen-in") citations pertinent to the reviewed topic, following protocol-defined criteria. In other words, they categorize, or label, citations as either "relevant" or "irrelevant" to the systematic review. Papers selected at this stage ("Level 1" screening) will be retrieved and appraised in full text ("Level 2" screening). In the modified approach we break "Level 1" screening into two phases. First, reviewers will train and use a classifier that categorizes citations as "relevant" or "irrelevant". They will trust the classifier in excluding completely "irrelevant" citations, and thus they will manually review only the citations that are screened in by the classification model. "Level 2" screening remains the same as in the typical approach.

#### Document (Citation) Representation

**Figure 2:**
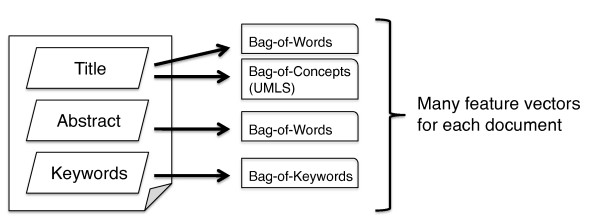
**An article is broken down into its component parts (title, abstract text, and keywords), and these are in turn represented as either bag-of-words or bag-of-UMLS-biomedical concepts vectors**.

The representation of biomedical documents can have a large effect on classification performance [8,13-15] and is arguably more important than the choice of the classification algorithm itself [22]. We represent each citation with points in four separate feature-spaces. In particular, we use the title text, the abstract text, MeSH keywords (when available) and UMLS terms (Figure 2). (We use the MetaMap [24] program to automatically extract UMLS terms from the title texts.) Over the texts, we use standard Term Frequency/Inverse Document Frequency (TF-IDF) encoding [25] to generate "bag-of-words" representations of each citation in the respective feature-spaces. Similarly, we generate a "bag-of-biomedical-terms" [8] representation in the UMLS feature-space. Other feature-spaces (e.g., the full text of each paper) could be used in addition to, or in place of, the four used here. We build an ensemble of SVM classifiers, with one classifier per feature space [8], and aggregate their predictions as described in the following subsections. (An alternative to this approach would be to join the feature-spaces into a single feature-space, concatenating the the respective points for a given document to form a single point in the combined space [10]. However, this combined representation can be problematic for active learning [26].)

#### Active Learning Strategy for Citation Classification

We have developed a novel active learning strategy for scenarios where classes are imbalanced and the costs of mistakes over these classes are asymmetric (e.g., false negatives in our case are more costly than false positives). The strategy first attempts to characterize the space of relevant citations before refining the decision boundary, or hyperplane, which separates "relevant" from "irrelevant" citations.

As discussed in the Background Section, uncertainty sampling active learning is an online training strategy that is more efficient than training on a random subset of data. In uncertainty sampling, the classifier requests labels for examples about whose class membership it is most uncertain, and in this way incrementally refines the current approximation to the separating boundary. With SVMs, this is equivalent to asking the reviewer to label citations nearest the (current) separating hyperplane [23]. In previous work we demonstrated that uncertainty sampling can result in classifiers with poor performance, in terms of sensitivity to the minority class, when classifying text documents [27]. Theoretically, this can happen if there are different clusters, or regions, of the minority class (here, "relevant" citations); in such a scenario, uncertainty sampling is prone to myopically focusing in on the first boundary or boundaries discovered, missing any other clusters. This problem of *hasty generalization *becomes particularly important when there are asymmetric misclassification costs, such as in citation screening, wherein the mistaken exclusion of an eligible paper is much more costly than wrongly including an ineligible paper.

Our algorithm, *Patient Active Learning (PAL)*, attempts to overcome the pitfall of hasty generalization in active learning. Briefly, our strategy works by first exploring the space of citations by requesting labels for randomly drawn instances until it is likely that a reasonable representation of the minority class has been encountered. Only then does the algorithm begin refining the current boundary via uncertainty sampling. The heuristic used to decide whether or not the space of minority examples has been adequately explored is a function of the diversity over the encountered minority examples thus far, as measured by the average angle between each pair in kernel-space [28]. In particular, when this value changes by less than or equal to ϵ for a pre-specified number of iterations, we assume the diversities have converged and switch to uncertainty sampling.

Typically, active learning frameworks have assumed only one underlying feature-space. However, we represent citations as points in multiple feature-spaces (see Figure 2) and build separate classifiers over each of these. Thus we need to adapt active learning to an ensemble (i.e., multiple-model) scenario. Moreover, we must address how to aggregate the predictions of the classifiers comprising this ensemble. Note that each feature-space will have its own corresponding separating hyperplane; thus we may be confident about the class membership of an example in one space while uncertain about the same example in another. It is therefore desirable to refine the boundaries in all *k *feature-spaces, rather than focusing on just one. Here we adopt the naive approach of picking a feature-space at random by flipping a fair, *k*-sided coin at each step in the active learning process, and requesting the label for the example closest to the hyperplane in this feature-space. Our approach to aggregating classifier predictions is similarly naive. Rather than outputting the majority vote of the *k *classifiers as the prediction for a particular unlabeled document, we predict "relevant" if *any *of the *k *classifiers predicts "relevant", due to our emphasis on sensitivity.

#### Class Imbalance and Aggressive Undersampling

Our datasets are (at times, extremely) imbalanced, i.e., the prevalance of "relevant" citations is always smaller than 50% (and often much smaller). Class imbalance presents a problem for classification algorithms, because they have typically been optimized for accuracy, rather than sensitivity to a particular class. Many techniques have been proposed to mitigate the effects of class imbalance and are reviewed at length elsewhere [29,30].

Here, we undersample (i.e., throw away instances from) the majority class (irrelevant citations) so that there are an equal number of labeled examples from each class prior to training our classifiers. This approach has been shown to work well with respect to increasing classifier sensitivity to the minority class [30]. We modify this strategy as follows: rather than undersampling the majority class at random, as is traditionally done, we throw out the majority examples nearest the current separating hyperplane. We call this *aggressive undersampling*. The idea is to explicitly push the decision boundary away from the minority class, as it has been observed that when there is class imbalance, SVMs are prone to discovering hyperplanes that are closer to the minority class than the ideal separating boundary, resulting in false negatives [31].

#### Summary of the Proposed Method

In review, our strategy is outlined in Algorithm 1, and is briefly summarized as follows. First, map each (initially unclassified) abstract into points in *k *different feature-spaces (here *k *= 4). Next, randomly pick abstracts for the reviewer to label until it is likely that the space of relevant citations has been explored (i.e., the diversity scores converge); at this point, switch to active learning via SIMPLE over a feature-space selected at random at each iteration. When some stopping criterion is satisfied (e.g., a pre-specified number of labels has been provided), aggressively under-sample the majority class (irrelevant abstracts) in each feature-space, then retrain and return an ensemble of SVMs, one per feature-space. Now, given an unlabeled abstract, d*, map d* into *k *feature vectors, and compute *k *predictions with the respective SVMs. If any of these predictions is "relevant", predict "relevant", else predict "irrelevant".

**Algorithm 1 **ScreenCitations(*D*)

**Input: ***D *- list of documents (citations) {*d*_1_, *d*_2_, ..., *d*_*N *_}

1. // First, pre-process the documents

2. Initialize *FS*_*l *_for each of the *k *feature-spaces to be used

3. **for ***d*_*i *_in documents **do**

4.    **for ***l *= 1 to *k ***do**

5.       // This step includes mapping free-text (e.g., title) to UMLS concepts

6.       Extract and encode a point *p *for *d*_*i *_in feature-space *l*

7.       Add *p *to *FS*_*l *_// Thus *FS_li _*is the *i*th document's point in feature-space *l*

8.    **end for **

9. **end for**

10.

11. // Now, start the training phase

12. Define (initially untrained) classifiers {*svm*_1_, *svm*_2_,..., *svm*_*k*_}

13. *U *← {1, 2, ..., *N*} // Put all the document indices in the "unlabeled pool" initially

14. *L *← {} // Initialize labeled pool

15. **while **Resources are available **do**

16.    **if **sufficiently explored the space of minority examples **then**

17.       // Pick the next example to label using SIMPLE over a randomly selected feature-space

18.       *l *← random number between 1 and *k*

19.       *i *← index of unlabeled document closest the hyperplane w.r.t. *svm*_*l*_

20.    **else**

21.       *i *← Random(*U*) // pick the next example to label at random

22.    **end if**

23.    // Here the reviewer would label *d*_*i*_

24.    *U *← *U*/*i*, *L *← *L *∪ *i*

25.    **for ***l *= 1 to *k ***do**

26.       *svm*_*l *_← train an SVM on examples {*FS_li _*for *i *∈ *L*}, using provided labels

27.    **end for**

28. **end while**

29.

30. // Finally, before outputting the final models, aggressively under-sample and re-train

31. *m *← ||labeled irrelevant citations|| - ||labeled relevant citations||

32. **for ***l *= 1 to *k ***do**

33.    // Remove the *m *majority examples closest to the hyperplane in this feature-space

34.    *L*_*undersampled *_← *L*/ closest *m *irrelevant citations w.r.t. *svm*_*l*_

35.    *svm*_*l *_← train an SVM on examples {*FS_li _*for *i *∈ *L*_*undersampled*_}

36. **end for**

37.

38. // Return the final models

39. **return **{*svm*_1_, *svm*_2_, ..., *svm*_*k*_}

end

### Experimental Setup and Results

In this section we first outline our experimental setup, including the datasets used, metrics considered and methodology employed. Next, we present our empirical results from experiments over three previously conducted systematic reviews.

#### Datasets

We experimented with datasets from three systematic reviews previously conducted by our team: the *Proton Beam *dataset [32], the chronic obstructive pulmonary disease (*COPD*) dataset (manuscript currently under review) and the *Micronutrients *dataset [33]. These are summarized in Table 1.

**Table 1:** In simulating the modified approach we considered as "relevant" the citations that were retrieved in full text ("Level 1" screening in Figure 1).

*Dataset*	*Total citations (N)*	*Retrieved in full text (% of N)*	*Included in the systematic review (% of N)*
Proton Beam	4,751	243 (5.1)	23 (0.5)
COPD	1,606	196 (12.2)	104 (6.5)
Micro Nutrients	4,010	258 (6.4)	139 (3.5)

The *Proton Beam *dataset is from a systematic review of comparative studies on charged particle radiotherapy *versus *alternate interventions for cancers [32]. The *COPD *dataset is from a systematic review and meta-analysis of all genetic association studies in chronic obstructive pulmonary disease. The *Micronutrients *dataset is from a systematic empirical appraisal of reporting of systematic reviews on associations of micronutrients and disease [33]. Note the class imbalance in all three datasets.

#### Evaluation of the Modified Approach

**Figure 3:**
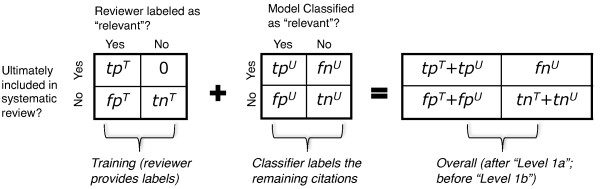
**Construction of confusion matrices for the semi-automated abstract screening strategy**. The leftmost matrix represents citations that are labeled by the reviewer while training the classification model. The middle matrix displays the predictions of the trained model over the remaining unlabeled set of citations *U*. The rightmost matrix shows the corresponding crosstabulation at the end of "Level 1a" (see Figure 1). The quantities mentioned in this figure are used in the definition of *Yield *and *Burden*, the chosen evaluation metrics (see Equations 1 and 2). Superscripts *T *and *U *refer to model training and applying the model to yet unlabeled citations, respectively. *tp*^[*T*|*U*]^: "true positives", *tn*^[*T*|*U*]^: "true negatives", *fp*^[*T*|*U*]^: "false positives", *fn *^[*T*|*U*]^: "false negatives". We assume that reviewers will never erroneously exclude a citation that is eligible for systematic review, i.e. *fn*^*T *^= 0.

Generally, classifiers are evaluated by considering their *predictive performance *as evaluated over a hold-out dataset. However, in this work we are not interested in predictive performance. We focus on whether a citation screening approach as a whole can identify all citations that are eventually eligible for the systematic review; we do not care if said citations are identified during training, or when the classifiers are applied to the unlabeled citations. To this end, we start our learners out with the same small initial set of labeled data, and then allow them to request labels for examples in the unlabeled pool, *U*. The center confusion matrix in Figure 3 shows the classifier predictions over the remaining unlabeled examples in *U*. To this we add the "relevant" and "irrelevant" citations labeled during training (the leftmost matrix) to produce the rightmost confusion matrix, used in the metrics defined below. Thus if an active learning strategy is somehow good at finding hard-to-classify examples in a pool during learning, and hence does not have to predict labels for these difficult instances, it is rewarded. We are concerned only with performance over the total pool of citations initially returned by the searches; anything else is immaterial for our purposes.

To make this point explicit, consider the following extreme scenario. Active learning strategies often ask for labels for a subset of citations that is enriched with "relevant" examples, compared to a random sampling. In our explorations the SIMPLE active learning strategy behaved this way. Assume an active learning strategy that happens to identify all abstracts that are finally eligible in the systematic review during training. We would definitely use this strategy, irrespective of its actual predictive performance; we do not care how it performs in a hold-out independent dataset, as long as it works well in the dataset at hand.

We introduce two metrics to evaluate citation screening approaches. The first metric, *Yield*, expresses the fraction of the citations that are finally eligible for the systematic review that are identified by employing a given citation screening approach. The second metric, *Burden *expresses the fraction of the total number of citations, *N*, that a human has to review manually with a given screening approach. In the typical approach reviewers screen manually the whole set of *N *citations. In the semi-automated approach they screen manually the citations that are presented to them during training, and only those suggested by the trained model as "relevant" (Figure 1). Figure 3 helps fix notation.(1)

In the typical approach of manual screening, both *Yield *and *Burden *are 100%. In the semi-automated approach the aim is to retain a *Yield *of 100% while minimizing the *Burden*.

#### Experimental Setup

We simulated the application of our semi-automated citation screening approach on datasets from three systematic reviews recently conducted by our team. We used two datasets, the *Proton Beam *and *COPD *datasets, during the development of our algorithm. From these we generalized a simple, operational stopping criterion for citation screening, i.e., a way of determining when enough labels have been provided to use the built classifier to classify the remaining citations. We kept the *Micronutrients *dataset as a holdout set to assess the generalizability of our approach and also to test our derived stopping criterion.

Experiments over the three datasets were conducted as follows. For each dataset, we initially hid all labels except for two; one citation from each class ("relevant" and "irrelevant") was selected at random and provided to the learning algorithm. (In practice systematic reviewers always know at least one relevant citation from the outset; indeed they often know of more than one, and including more relevant citations from the start would likely expedite training.) We then simulated active learning by allowing the learning algorithm to pick a number of citations to label at each step (we used 5 in our experiments, arbitrarily). The labels of the selected examples were revealed to the learner and subsequently used in training, and the examples were then removed from the unlabeled pool, *U*. Every 25 labels, we evaluated the current classifier over the remaining examples in *U*, calculating *Yield *and *Burden *as outlined in the above section. We continued active learning until *U *was exhausted, i.e., until all citations had been labeled. This whole process was repeated 10 times, and we report the averages over the runs.

#### Empirical Results

**Figure 4:**
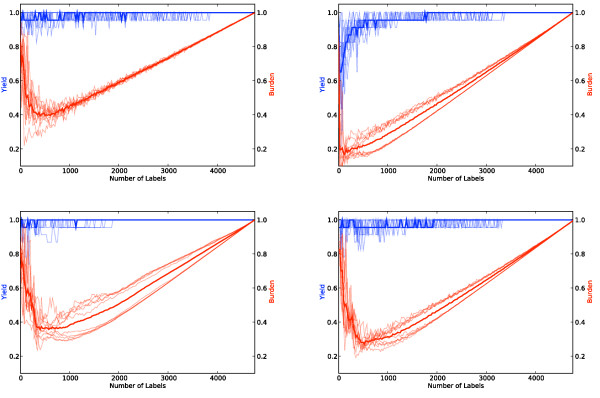
**Yield (blue) and burden (red) curves for four learning strategies over the proton beam dataset as a function of the size of thetraining set**. The thick lines are averages over 10 runs. Thin lines denote individual runs. Clockwise from the upper left, the strategies shown are: random sampling, SIMPLE, *PAL*, and *PAL *with aggressive undersampling. It is desirable to achieve maximum *Yield *while minimizing *Burden*. The upper right-corner (100% yield and 100% burden) corresponds to the manual approach of citation screening. Every point where *Yield *(the blue line) is at 1.0 and *Burden *(the red line) is less than 1.0 is thus progress. Note that *Burden *curves are U-shaped because classifiers trained on very small training sets tend to classify the majority of the unlabeled citations as "relevant" (due to our undersampling and cautious aggregation technique), and all citations classified as "relevant" must be subsequently screened by a human. When the training set is very large, the reviewers manually screen the majority of the citations during training.

We first motivate the use of our variant of active learning, *PAL*, and aggressive under-sampling (see Algorithm 1) by presenting results over the *Proton Beam *dataset for four different active learning strategies: naive random sampling (equivalent to passive learning), classical SIMPLE, *PAL *and *PAL *with aggressive undersampling. All of the approaches shown use the same four feature-spaces (see Figure 2) and a linear kernel SVM. (We used linear kernels because our data is high-dimensional [34]. We kept *C *set to its default value of 1 and did not perform parameter tuning, as we found its affect on performance to be negligible, possibly due to the sparseness of the feature space.) In all but *PAL *with aggressive undersampling we use standard undersampling, i.e., we throw away labeled irrelevant abstracts at random to achieve an equal class distribution prior to training the final classifier. In Figure 4, one can see that *PAL *with aggressive under-sampling (Algorithm 1) performs the best over this dataset. If the reviewer labels 2,000 abstracts, this method reduces their *Burden *by a bit less than half, on average. Moreover, not once were any of the 23 truly relevant citations misclassified with this method.

**Figure 5:**
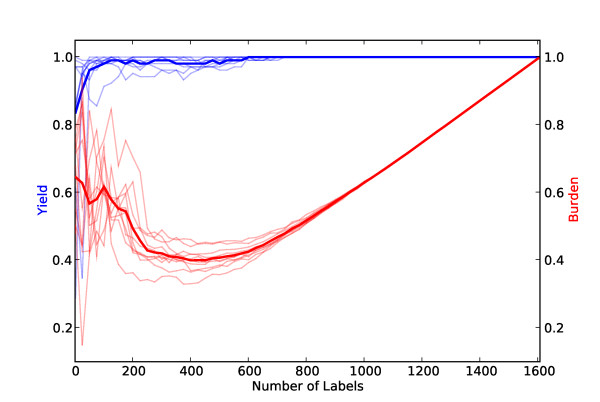
**Results over the COPD dataset**.

Over the *COPD *dataset, our citation screening approach could have reduced the burden on reviewers by approximately 40% while maintaining 100% sensitivity to the relevant citations, as can be seen in Figure 5, at the 750-800 label mark. We omit the other algorithms because *PAL *with aggressive undersampling again performed best. This dataset is considerably smaller, which likely explains the lower burden reduction compared to the results over the *Proton Beam *dataset. (We hypothesize that semi-automated citation screening will be most useful on large datasets.)

**Figure 6:**
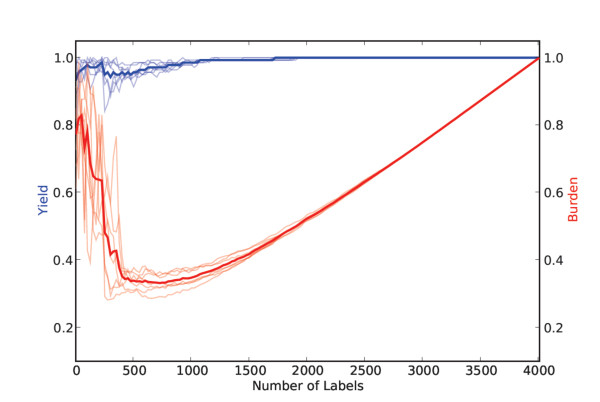
**Results over the micro-nutrients dataset**.

We extrapolated a simple operational stopping criterion from the results over the *Proton Beam *and *COPD *datasets: we hypothesized that training on half of the dataset would be sufficient to achieve 100% *Yield*. The results over the *Micronutrients *dataset are shown in Figure 6. Using our simple stopping criterion of stopping after half of the citations (2,000, in this case) are labeled would have reduced the *Burden *on reviewers by nearly half, on average, while maintaing 100% *Yield*.

## Discussion

We managed to accomplish our goal of reducing the number of abstracts that would have needed to be manually screened by nearly 50% without missing any relevant abstracts on the *Proton Beam *dataset. Likewise, over the *COPD *systematic review data, our method would have reduced the *Burden *on the reviewers by ~ 40%, on average, again without missing any relevant citations. However, our strategy was in a sense 'optimized' for these two systematic reviews, because they were used to evaluate and tune our approach (choice of document representation, classification and re-sampling algorithms, etc.) during testing and development. Regardless, the fact that our technique performed so well over ten independent runs on these real-world datasets is encouraging.

From the *COPD *and *Proton Beam *datasets we extrapolated a simple stopping criterion, i.e., a minimum number of citations that need to be labeled for training before applying the classifier to the remaining unlabeled citations. This is an important practical issue in deploying a system for semi-automating the citation screening process. If no such stopping criterion is provided, reviewers will have no way of knowing how many citations they must manually screen. We observed that in both the *Proton Beam *and *COPD *datasets *Yield *was consistently 100% after half of the citations were labeled (see Figures 4 and 5). We thus adopted this - labeling half of the citations - as our hypothetical stopping criterion for the *Micronutrients *dataset. Our results over this hold-out dataset, which we did not experiment with during the development of our algorithm, satisfied our stated aim of achieving 100% *Yield *while significantly reducing the *Burden *using our simple stopping criterion.

These initial results are promising, but there is room for improvement. In particular, while we achieved our aim of achieving perfect sensitivity to relevant abstracts, the *Burden *remains rather high at 50 to 60%. Ideally, we could reduce this burden while maintaining perfect *Yield*. We are optimistic about the prospects of further improving our method. Many sources of information remain to be exploited. For example, we plan on incorporating more feature sets, including full document text, citation networks, and so forth. Moreover, we plan on further enriching the features that we are using, e.g. by extracting UMLS concepts from the abstract text. Other encoded ontologies might also provide a source of enrichment [35].

Aside from new feature sets, a few algorithmic improvements may improve performance. One obvious technique that we plan on implementing is bagging [36], in which multiple subsets of each feature-space would be constructed at random from the labeled data, and used to build ensemble classifiers over each feature-space. Another possible improvement would be to follow the suggestion of Kilicoglu et al. and employ different classification algorithms over the different feature-spaces [8] (here we used only SVMs). We are also interested in the emerging work on active learning over *features *rather than instances [37], which may be helpful in identifying phrases or UMLS concepts particularly characteristic of relevant citations. Indeed, active learning over features may provide a novel framework for extracting and modeling the reviewer's expertise. Finally, exploiting latent hierarchical structure in the UMLS ontologies might also be helpful, particularly because active learning techniques for hierarchical data have recently been developed [38].

Our results here are promising, but more extensive testing remains to be done. We plan on assembling 20-30 systematic review datasets for use in a large-scale validation of our method.

## Conclusions

We have presented a strategy for semi-automating the laborious, tedious task of citation screening for systematic reviews, and provided evidence that our method can significantly reduce reviewers' workloads. The burden on researchers undertaking systematic reviews is only going to increase with the exponentially growing body of biomedical literature. This work is a step towards alleviating a large part of this burden without sacrificing the scientific thoroughness of conducted reviews.

## Methods

Our code is written in Python and makes use of a modified version of LibSVM [39]. Abstract and title texts were pre-processed by removing stop words from the pubmed stop-word list [40], and also removing all words that appeared fewer than 3 times (this was an arbitrary number picked to reduce feature-space size).

To extract UMLS concepts, we used the MetaMap transfer application [24] (available at http://www.nlm.nih.gov/research/umls/mmtx.html.) Using this program, a list of all discovered UMLS terms was generated for each abstract title in the whole set of abstracts. We then mapped title texts to a bag-of-biomedical concepts representation, treating the UMLS terms as words.

We fully plan on open-sourcing our abstract screening code once it is further validated (and documented). Indeed, we have included the source code used in experimentation as additional file 1 (curious snake.zip). We also plan to eventually make our systematic review datasets publically available, to allow other researchers to work with them. In the meantime, if a researcher is interested in obtaining the current version of the code (or datasets), he or she may contact the authors.

## Authors' contributions

BCW wrote the first draft of the paper that was critically commented on by all authors. BCW, CB and TAT developed algorithms. BCW and TAT assembled the databases. BCW implemented software code and performed. Findings were interpreted by all authors. All authors have read and approved the final manuscript.

## Supplementary Material

Additional file 1 **Curious snake: a zipped archive of source code**. This is the source code used in experimentation. It is a modified version of our Python-based active learning framework. Click here for file

## References

[B1] Barza M Trikalinos TA Lau J Statistical considerations in meta-analysis Infect Dis Clin North Am 2009 23 195 210 10.1016/j.idc.2009.01.003 19393905

[B2] Counsell C Formulating questions and locating primary studies for inclusion in systematic reviews Ann Intern Med 1997 127 380 387 9273830 10.7326/0003-4819-127-5-199709010-00008

[B3] Wheeler P Balk E Bresnahan K Shephard B Lau J DeVine D Chung M Miller K Criteria for determining disability in infants and children: short stature Evidence Report/Technology Assessment No. 73. Prepared by New England Medical Center Evidence-based Practice Center under Contract No. 290-97-001 2003 PMC4781540 12749119

[B4] Cole C Binney G Casey P Fiascone J Hagadorn J Kim C Wang C Devine D Miller K Lau J Criteria for determining disability in infants and children: Low Birth Weight Evidence Report/Technology Assessment No. 70. Prepared by New England Medical Center Evidence-based Practice Center under Contract No. 290-97-0019 2002 PMC4781106 15523747

[B5] Perrin E Cole C Frank D Glicken S Guerina N Petit K Sege R Volpe M Chew P MeFadden C Devine D Miller K Lau J Criteria for determining disability in infants and children: failure to thrive Evidence Report/Technology Assessment No. 72. Prepared by New England Medical Center Evidence-based Practice Center under Contract No. 290-97-0019 2003 PMC4781624 12749118

[B6] Hunter L Cohen KB Biomedical Language Processing: What's Beyond PubMed? Mol Cell 2006 21 5 589 594 10.1016/j.molcel.2006.02.012 16507357 PMC1702322

[B7] Yu W Clyne M Dolan SM Yesupriya A Wulf A Liu T Khoury MJ Gwinn M GAPscreener: An automatic tool for screening human genetic association literature in PubMed using the support vector machine technique BMC Bioinformatics 2008 205 9 10.1186/1471-2105-9-205 PMC2387176 18430222

[B8] Kilicoglu H Demner-Fushman D Rindflesch TC Wilczynski NL Haynes BR Towards Automatic Recognition of Scientifically Rigorous Clinical Research Evidence J Am Med Inform Assoc 2009 16 25 31 10.1197/jamia.M2996 18952929 PMC2605595

[B9] Chen D Muller HM Sternberg PW Automatic document classification of biological literature BMC Bioinformatics 2006 7 370 10.1186/1471-2105-7-370 16893465 PMC1559726

[B10] Aphinyanaphongs Y Tsamardinos I Statnikov A Hardin D Aliferis CF Text categorization models for high-quality article retrieval in internal medicine Journal of the American Medical Informatics Association: JAMIA 2005 12 2 207 216 10.1197/jamia.M1641 15561789 PMC551552

[B11] Aphinyanaphongs YAC Text Categorization Models for Identifying Unproven Cancer Treatments on the Web Medinfo 2007: Proceedings of the 12th World Congress on Health (Medical) Informatics 2007 968 972 17911859

[B12] Blum A Mitchell T Combining Labeled and Unlabeled Data with Co-Training 1998 Morgan Kaufmann Publishers 92 100

[B13] Blake C Pratt W Better Rules, Fewer Features: A Semantic Approach to Selecting Features from Text 2001 ICDM, San Jose, CA 59 66

[B14] Wilcox A Hripcsak G Friedman C Using Knowledge Sources to Improve Classification of Medical Text Reports KDD-2000 Workshop on Text Mining (poster) 2000 Boston, MA

[B15] Yetisgen-Yildiz M Pratt W The Effect of Feature Representation on MEDLINE Document Classification AMIA Symposium Proceedings 2005 PMC1560754 16779160

[B16] Cohen A Hersh W Peterson K Yen PY Reducing Workload in Systematic Review Preparation Using Automated Citation Classification J Am Med Inform Assoc 2006 13 206 219 10.1197/jamia.M1929 16357352 PMC1447545

[B17] Lewis DD Gale WA A sequential algorithm for training text classifiers SIGIR '94: Proceedings of the 17th annual international ACM SIGIR conference on Research and development in information retrieval 1994 New York, NY, USA: Springer-Verlag New York, Inc 3 12

[B18] Schohn G Cohn D Improving Generalization with Active Learning Proc 17th International Conf on Machine Learning 2000 San Francisco, CA: Morgan Kaufmann 839 846

[B19] Settles B Active Learning Literature Survey Tech Rep 1648 2009 University of Wisconsin-Madison

[B20] Vapnik VN The nature of statistical learning theory 1995 New York, NY, USA: Springer-Verlag New York, Inc

[B21] Joachims T Text categorization with Support Vector Machines: Learning with many relevant features ECML 1998 137 142 full_text

[B22] Zweigenbaum P Demner-Fushman D Yu H Cohen KB Frontiers of biomedical text mining: current progress Briefings in Bioinformatics 2007 8 5 358 375 10.1093/bib/bbm045 17977867 PMC2516302

[B23] Tong S Koller D Support Vector Machine Active Learning with Applications to Text Classification Proceedings of the Seventeenth International Conference on Machine Learning 2000 999 1006

[B24] Aronson A Effective Mapping of Biomedical Text to the UMLS Metathesaurus: The MetaMap Programjournal of biomedical informatics formerly computers and biomedical research 2001 35 17 PMC2243666 11825149

[B25] Jones KS A statistical interpretation of term specificity and its application in retrieval Journal of Documentation 1972 28 11 21 10.1108/eb026526

[B26] Goh KS Chang EY Lai WC Multimodal concept-dependent active learning for image retrieval MULTIMEDIA '04: Proceedings of the 12th annual ACM international conference on Multimedia 2004 New York, NY, USA: ACM 564 571 full_text

[B27] Wallace B Trikalinos T Lau J Schmid C Brodley C SRSM (Presentation), Seattle, WA 2009

[B28] Brinker K Incorporating diversity in active learning with support vector machines Proceedings of the 20th International Conference on Machine Learning 2003 AAAI Press 59 66

[B29] Kotsiantis S Kanellopoulos D Pintelas P Handling imbalanced datasets: A review GESTS International Transactions on Computer Science and Engineering 2006 30 25 36

[B30] Japkowicz N Learning from Imbalanced Data Sets: A Comparison of Various Strategies AAAI 2000 10 15

[B31] Ertekin S Huang J Giles LC Active learning for class imbalance problem SIGIR '07: Proceedings of the 30th annual international ACM SIGIR conference on Research and development in information retrieval 2007 New York, NY, USA: ACM 823 824 full_text

[B32] Terasawa T Dvorak T Ip S Raman G Lau J Trikalinos TA Charged Particle Radiation Therapy for Cancer: A Systematic Review Ann Intern Med 2009 556 565 19755348 10.7326/0003-4819-151-8-200910200-00145

[B33] Chung M Balk EM Ip S Raman G Yu WW Trikalinos TA Lichtenstein AH Yetley EA Lau J Reporting of systematic reviews of micronutrients and health: a critical appraisal Am J Clin Nutr 2009 89 1099 1113 10.3945/ajcn.2008.26821 19244363 PMC2667458

[B34] Chih-Wei Hsu CJL Chih-Chung Chang A Practical Guide to Support Vector Classification Tech rep 2000

[B35] Camous F Blott S Smeaton AF Hochreiter S, Wagner R, Hochreiter S, Wagner R Ontology-Based MEDLINE Document Classification BIRD, of Lecture Notes in Computer Science 2007 4414 Springer 439 452 full_text

[B36] Breiman L Breiman L Bagging Predictors Machine Learning 1996 123 140

[B37] Druck G Settles B McCallum A Active Learning by Labeling Features Proceedings of the Conference on Empirical Methods in Natural Language Processing (EMNLP) 2009 81 90

[B38] Dasgupta S Hsu D Hierarchical sampling for active learning ICML '08: Proceedings of the 25th international conference on Machine learning 2008 New York, NY, USA: ACM 208 215 full_text

[B39] Chih-Chung Lin CJ LIBSVM: a library for support vector machines 2001 http://www.csie.ntu.edu.tw/~cjlin/libsvm/

[B40] PubMed Stopword List 2009 http://www.ncbi.nlm.nih.gov/entrez/query/static/help/pmhelp.html#Stopwords

